# Genotypic and Phenotypic Modifications of *Neisseria meningitidis* after an Accidental Human Passage

**DOI:** 10.1371/journal.pone.0017145

**Published:** 2011-02-28

**Authors:** Hélène Omer, Graham Rose, Keith A. Jolley, Eric Frapy, Jean-Ralph Zahar, Martin C. J. Maiden, Stephen D. Bentley, Colin R. Tinsley, Xavier Nassif, Emmanuelle Bille

**Affiliations:** 1 INSERM U1002, Paris, France; 2 Université Paris Descartes, Faculté de Médecine Necker-Enfants Malades, Paris, France; 3 The Wellcome Trust Sanger Institute, Hinxton, Cambridge, United Kingdom; 4 Department of Zoology, University of Oxford, Oxford, United Kingdom; 5 Assistance publique - Hôpitaux de Paris, Laboratoire de Microbiologie, Hôpital Necker-Enfants Malades, Paris, France; 6 Genetic regulation and biofilms, UMR Micalis 1319, INRA/AGROPARITECH, Thiverval-Grignon, France; Institut de Pharmacologie et de Biologie Structurale, France

## Abstract

A scientist in our laboratory was accidentally infected while working with Z5463, a *Neisseria meningitidis* serogroup A strain. She developed severe symptoms (fever, meningism, purpuric lesions) that fortunately evolved with antibiotic treatment to complete recovery. Pulse-field gel electrophoresis confirmed that the isolate obtained from the blood culture (Z5463BC) was identical to Z5463, more precisely to a fourth subculture of this strain used the week before the contamination (Z5463PI). In order to get some insights into genomic modifications that can occur *in vivo*, we sequenced these three isolates. All the strains contained a mutated *mutS* allele and therefore displayed an hypermutator phenotype, consistent with the high number of mutations (SNP, Single Nucleotide Polymorphism) detected in the three strains. By comparing the number of SNP in all three isolates and knowing the number of passages between Z5463 and Z5463PI, we concluded that around 25 bacterial divisions occurred in the human body. As expected, the *in vivo* passage is responsible for several modifications of phase variable genes. This genomic study has been completed by transcriptomic and phenotypic studies, showing that the blood strain used a different haemoglobin-linked iron receptor (HpuA/B) than the parental strains (HmbR). Different pilin variants were found after the *in vivo* passage, which expressed different properties of adhesion. Furthermore the deletion of one gene involved in LOS biosynthesis (*lgtB*) results in Z5463BC expressing a different LOS than the L9 immunotype of Z2491. The *in vivo* passage, despite the small numbers of divisions, permits the selection of numerous genomic modifications that may account for the high capacity of the strain to disseminate.

## Introduction

*Neisseria meningitidis* (*Nm*) is primarily an asymptomatic coloniser of the human nasopharynx of about 10% of the population [1]. But it is better known as the causative agent of meningococcal disease, which kills an estimated 50,000 individuals worldwide annually [2]. In these cases, *Nm* disseminate from the nasopharynx into the bloodstream where they can survive and divide giving a bacteraemia, which can evolve to meningitis or septicaemia. The meningococcal population is classified in different phylogenetic groups, the clonal complexes, defined by multilocus sequence typing (MLST; [3]). The large majority of disease is caused by a small number of these groups, which are hence called hypervirulent clonal complexes [3]. In industrialised countries, most neisserial disease is caused by serogroup B and C strains whereas serious epidemics occur in the sub-Saharan part of Africa due to serogroup A strains [4]. It is worth noticing the emergence of the serogroups X, W135 and Y worldwide.

The reasons for the occurrence of disease in some individuals but not in others remain unclear, even if both human and bacterial factors have been implicated in the different possible outcome of the infection. The most important human factors to counter neisserial disease are the bactericidal antibodies [5], [6] and the complement system [7]. Among the bacterial factors, the protective polysaccharide capsule is necessary for survival in the bloodstream and subsequent disease, even if some acapsulated bacteria have recently been isolated from patients [8], [9]. Several other bacterial structures are important for bacterial pathogenesis: the endotoxin lipooligosaccharide (LOS; [10]), the type IV pili [11], the recently described proteins capable of binding complement factor H (FhbP, NspA) [12], [13], and the iron acquisition systems from transferrin and lactoferrin (transferrin binding protein TbpA and TbpB; [14]) or haemoglobin and haptoglobin (HpuAB and HmbR) [15]–[17]. By comparing the genomes of meningococci belonging to clonal complexes designated as having higher or lower invasive potential [18], [19], a prophage was shown to be present at higher frequency in strains responsible for invasive disease. This genetic element was designated MDA for Meningococcal Disease-Associated island [20]. Subsequent analyses have demonstrated that a fraction of the invasiveness of strains belonging to hyperinvasive clonal complexes is correlated with the presence of the prophage, and showed an association with virulence in young adults but not in children less than two years of age [21]. However the mechanism by which this prophage increases invasiveness remain unclear. Beside the insertion of this prophage, no known genomic island has been associated with invasiveness. Indeed, many meningococci belonging to clonal complexes that are not hyperinvasive have all the necessary attributes to survive in the extracellular fluids. Furthermore, established or potential virulence factors are not universally present in meningococci isolated from invasive disease.

The ability of the bacteria to adapt to changing environments is key for invasiveness. Some of the neisserial virulence attributes are tightly controlled at the transcriptional level by regulators such as the Fur protein responding to variations in iron concentration [22], or by two component-systems such as PhoP(MisR)/PhoQ(MisS) [23]. In addition, several of the bacterial attributes required for meningococcal colonization and/or dissemination are subject to phase and/or antigenic variation [24]–[29], thus allowing the bacteria to rapidly adapt to its environment. The genomic modifications responsible for phase variation occur at a higher frequency in hypermutator strains defective in post-replication DNA mismatch correction, most often due to mutations in the repair genes *mutS*, *mutL* or *mutY*. Some pathogenic strains from epidemics have been shown to display this kind of hypermutator phenotype especially during the African epidemics due to serogroup A strains [30], [31].

As for many human specific diseases, the difficulty of reproducing the human disease in animal models has hampered the identification of the neisserial attributes required for invasiveness. Several studies have reported clinical laboratory acquired meningococcemia [32]–[38]. These reports focussed on tracing the infecting strain amongst those manipulated by the patient during the weeks before the disease. Here we took advantage of a laboratory contamination responsible for an invasive meningococcemia to compare the genome of the bloodstream isolate with that of the contaminating isolate. The bloodstream isolate differed from the infecting strain mostly due to phase variation of virulence genes allowing the expression of different phenotypes. Amongst these phenotypes are the ability of the strain to use different receptors to get iron from the host haemoglobin, the expression of a different LOS to evade the host immune system and the presence of different pilin variants conferring different properties on human cells. Our data showed that, despite the small number of *in vivo* generations between the contaminating and the bloodstream isolates, limited genomic modifications have been selected that may account for the ability of the strain to increase its invasiveness.

## Materials and Methods

### Clinical case

In November 2007, a 32 years-old female working in our laboratory was admitted in the infectious diseases unit at the Necker-Enfants Malades Hospital (Paris, France) with fever, meningism and small purpuric lesions. The night before admission, she complained of fever, chills and headache. Upon admission, she was alert and orientated but suffered from severe headache and was febrile at 39°C. Her physical examination revealed nuchal rigidity with small purpuric lesions on all the four limbs. Antibiotic treatment (intravenous ceftriaxone 50 mg/kg/day) was administered just after a blood culture sampling. A lumbar puncture revealed a clear cerebrospinal fluid (CSF). CSF protein was 0.84 g/L, glucose 2.6 mmol/L with blood glucose at 5.6 mmol/L. CSF contained 1,190/mm^3^ red blood cells and 90/mm^3^ white blood cells (90% PNN) and no germ was detectable on Gram stain. A non-contrast CT-scan was normal. The next day, blood culture was positive for serogroup A meningococci. Ceftriaxone was carried on for 7 days. The patient state improved rapidly and she was discharged on day 5 without sequellae.

The patient had no apparent immune defect (notably, no complement anomaly was detected) or known predisposing factor for meningococcal disease. She had been working at the bench with *Nm* for five years and had therefore been vaccinated in October 2002 with the meningococcal A + C polyosidic vaccine (Sanofi Pasteur MSD). This vaccine is efficient for 3 years; no booster had been administered. A thorough investigation of the research practices and the material were performed and revealed that the accidental contamination was due to the malfunction of a hood.

According to the french law and considering that this study was not an experimental study in humans, this study did not need approval by an ethic committee. Written informed consent from the patient was obtained to perform this study and to publish the data as presented in this manuscript.

### Bacterial strains and growth medium

Because *Nm* Z2491 (formerly C751), whose genome has been completed by the Sanger centre [25], is not transformable, we are routinely working with Z5463 (formerly C396). Z2491 and Z5463 were isolated during the same epidemic in The Gambia in 1983 [39]. Z5463 is a naturally transformable serogroup A strain belonging to the same sequence type as strain Z2491, i.e. ST-4 [40], subgroup IV-1, expressing OpaA and OpaC. This strain was stored frozen at −80°C and was used at that time to explore the role of the MDA phage in invasiveness. The person who was contaminated worked on isolating spontaneous variants of Z5463 that expressed high levels of the MDA phage proteins (see below). The week before the contamination she was working with such a derivative of Z5463 designated Z5463PI (for Plate Isolate). Four passages took place between the frozen stock of Z5463 and the frozen stock of Z5463PI. The strain isolated from the bloodstream was designated Z5463BC (for Blood Culture). The blood culture was directly frozen at the hospital without further subculture. In order to avoid as much as possible secondary variations, all the experiments have been carried out on direct cultures of frozen stocks.

*Neisseria* were routinely grown at 37°C in 5% CO_2_ in GC-liquid medium or on GC medium base (Difco) containing Kellogg's supplements [41]. When needed, antibiotics were used at a concentration of 5 mg/mL of chloramphenicol, 200 mg/mL of kanamycin and 75 mg/mL of spectinomycin. The frequency of occurrence of rifampicin resistance mutants was obtained by plating bacteria onto GCB agar plates supplemented or not with 30 mg/mL of rifampicin.

To test the ability of *Nm* to chelate iron from haemoglobin, a single colony taken from an overnight growth on agar plate was resuspended in liquid GCB and plated onto GCB agar supplemented with 12 mM ferric nitrate or 50 mM deferoxamine mesylate (Sigma). The medium was allowed to evaporate for approximately 15 min and discs containing either 50 mg of ferric nitrate or 100 mg of human or bovine haemoglobin (Sigma) were placed on the plate.

### Pulsed field gel electrophoresis and detection of the MDA phage by Southern-blot

Bacteria were suspended in PBS - 7% sucrose to an OD600 of 1, mixed with an equal volume of 2% Agar Low-Melting-Point and child in a plastic mould. Once the plugs were solid, bacterial proteins were lysed by the addition of EDTA (0.5 M), SDS 1% and proteinase K (0.5 mg/mL) at 56°C for 24 h. The reaction was stopped by the addition of PMSF and the plugs were washed with TE. The plugs were equilibrated in 300 µL of digestion buffer for 1 h before a 24 h incubation at 37°C with 20 units of *Bgl*II (New England BioLabs). The plugs were then cast in a 1% agarose gel. Electrophoresis was performed on a Chef-mapper (Bio-Rad) at 14°C, at 6 V/cm with pulses ramping from 0.5 to 30 sec over 20 h with an angle of 120°. The gel was stained with ethidium bromide and photographed under UV light.

DNA was transferred from the gel to a nylon membrane Hybond N+ (Amersham) by capillary action and fixed by a 5 min exposition to UV light. The membrane was then hybridized to γ-^33^P dCTP labelled PCR product of *NMA1792* [20] to detect the MDA insertions. Bound probes were detected by exposure of the membrane overnight at −20°C between intensificating screens. The screen was read on a Storm 840 (GE Healthcare) with a 50 pixels resolution.

### Search for chromosomal rearrangements induced by the MDA phage

We have previously described that the MDA prophage is inserted in dRS3 repeats [20], more precisely in some target dRS3 sequences (consensus dRS3/Nf1 sequence: ATTCCCgcCTgcgcGGGAAT; [42]). These sequences are present 291 times in the genome of Z2491 (672 dRS3 sequences in total) and assembled in 89 “neisserial intergenic mosaic elements” (NIME) that correspond to dRS3 sequences flanked by RS sequences [25]. To detect putative chromosomal rearrangements induced by the MDA, we designed PCR primers to discriminate each of these 89 NIME (Table S1). Total DNA was extracted using the Wizard Genomic DNA purification kit (Promega) and PCR was performed using Ex-Taq (Takara) and pairs of the above primers. PCR were performed to amplify independently each NIME. The presence and size of every band was assessed to verify that the chromosomal arrangement was according to the known genome sequence. To identify possible rearrangement occurring between two NIME arrays, each primer specific of a NIME was tested against all the other primers.

### Mapping of the MDA insertions

To precisely map the phage chromosomal insertions, Ligation-Mediated PCR were performed as described by Pelicic and co-workers [43]. Total DNA was digested with *Nae*I or *Mbo*I (New England Biolabs). The DNA was ligated with the linkers LMP1, LMP2 or LMP3. Primers corresponding to the MDA (MDAbeginR and MDAendF) were used with the appropriate linker to amplify and sequence the sites of insertion of the MDA (Table S1).

### Detection of the production of the MDA phage

The production of MDA phage was assessed by the detection of circular form of the MDA DNA by PCR, using MDAbeginR and MDAendF as primers (Table S1).

A bioinformatic analysis of the ORFs encoded by the MDA prophage identified NMA1796 as the putative major capsid protein. A polyclonal anti-NMA1796 antibody was raised and purified using the following peptide: H_2_N-CINFLKDMGKVGTD-COOH. The analysis of the production of phage proteins was performed by immunoblot directly on colonies after transfer onto a nitrocellulose filter. Briefly, isolated colonies grown overnight were transferred to a reinforced nitrocellulose membrane (0.2 mm pore size, Optitran BA-S 83) and allowed to air dry. Non-specific binding sites were blocked for 1 h with PBS - 5% milk. Filters were washed and incubated for 1 h with a 10,000-fold dilution of antiserum raised against the peptide of NMA1796. Excess antibody was removed by washes in PBS-Tween and the filters were incubated for 1 h with an anti-rabbit IgG-peroxydase antibody (Sigma). After washing, bound antibodies were detected by chemiluminescence using ECL plus reagents (GE Healthcare).

### Engineering of Nm mutants

A mutation in *hpuA* (Δ*hpuA*) was engineered by introducing the chloramphenicol resistance cassette in the gene. The *hmbR* mutation (Δ*hmbR*, spectinomycin resistant) and the *pilE* mutation (Δ*pilE*, kanamycin resistant) have been previously described ([44], [45], respectively). These mutations were transformed into Z5463, Z5463PI or Z5463BC as needed. When necessary, *mutS* from either Z5463 or Z2491 was cloned and a spectinomycin resistance cassette was inserted after the gene, leaving a downstream intergenic sequence at the 3′-end, thus allowing to perform the allelic exchange. All mutants were checked using PCR amplification and sequencing. Primers are listed in Table S1.

### LOS extraction

Meningococcal LOS was purified from total cell extracts of overnight plate by a proteinase K treatment as previously described [45]. The size differences were observed on a 16% Tricine SDS-PAGE gel by silver staining.

### RNA extraction and transcriptomic studies

RNA was extracted by a Trizol chloroform method before using the RNeasy clean up protocol from Qiagen. DNA was removed by addition of Turbo DNaseTM (Ambion). The complete removal of DNA was assessed by the absence of signal in a PCR run.

Equal amounts of RNAs of each strain were labeled differentially using Cy3-dCTP and Cy5-dCTP (Amersham Pharmacia) during a first reverse-transcriptase step using the Superscript II RNase H− reverse transcriptase (Life Technologies) and a balanced mixture (20 pmol each) of C-terminal primers specific for each gene present on the microarrays. The differentially labeled cDNA were combined and hybridized overnight at 42°C to the *Neisseria* chips (Eurogentec), composed of overlapping 40-mer oligonucleotides covering the entire genome of strain Z2491. After washing, arrays were scanned using the Axon 4000B Scanner (Genepix Pro) with the Limma Bioconductor program. The fluorescence data were normalized and the fluorescence ratios were calculated from the normalized values. Each experiment was repeated three times in swap dye, genes were referred as being differentially transcribed when a minimal 2-fold deregulation on average in more than 50% of the independent experiments was observed.

### Cell adhesion and signalisation assays

The immortalized human brain microvessel endothelial cell line (hCMEC/D3) [46] was grown and infected as described by Coureuil and co-workers [47]. Briefly the cells were infected at a multiplicity of infection of 100. For cell adhesion assays, after extensive washing to remove unbound *Nm*, the human cells were scraped from the wells, and the number of cell associated CFUs determined.

When needed, immunofluorescence assays were performed after 2 h of infection. Antibodies and markers used included: rabbit anti-Ezrin polyclonal antibody (1∶1,000), generously provided by Dr. P. Mangeat (CNRS, UMR5539, Montpellier, France) and Alexa-488 conjugated anti-rabbit IgG secondary antibody (1∶200, Invitrogen); Alexa-546 conjugated phalloidin and DAPI (0.5 µg/mL, Sigma).

### Genome sequencing and analysis

Genome sequencing, data collection and first analyses were performed at the Wellcome Trust Sanger Institute. Genome sequences were generated by whole-genome shotgun sequencing (454 Life Science, Inc.) of Z5463, Z5463PI and Z5463BC giving a 35-, 23- and 26-fold coverage, and by Illumina GA sequencing (Solexa) of Z5463, Z5463PI and Z5463BC giving a 154-, 84- and 78-fold coverage. The genomes were assembled using MAQ (Mapping and Assembly with Qualities; [48]) with Z2491 sequence [25] as the assembly reference genome. The sequences are deposited in the PubMLST website ([Neisseria PubMLST:17881–17883]).

All the following analyses were made using Z2491 sequence as the reference genome. The MAQ program was used to detect and extract all the Single Nucleotide Polymorphism (SNP) between the reference and each strain. The SNP were extracted, automatically validated and then assessed manually, when we were unable to conclude the region was again sequenced (see below). For broader genome rearrangement, we used the programs ABACAS [49] and Artemis [50]. The presence of numerous repeats made it difficult to discriminate some contig layouts. Primers discriminating between every set of NIME were used as previously described. We focused on these repeats because (i) they were often associated with inconclusive layouts, (ii) they have already been suggested to allow genetic recombination between strains [42], [51] and (iii) the Z5463PI and Z5463BC strains contain a second insertion of the MDA phage that specifically insert in specific dRS3 repeats in NIME sequences [20].

PCR were performed for each inconclusive sequence (sequence lacking or discrepancy) using the two methods (454 and Solexa) and sequenced using standard dye-terminator technology. Primers used for these amplifications have been previously described (Perrin *et al.*, 2002). The sequence verifications were done using the Big Dye Primer Sequencing Kit (Applied Biosystems) and read on an ABI-Prism 310 automated sequencer (Applied Biosystems).

## Results

### Duplication of the MDA prophage in the infecting strain

**Figure 1:**
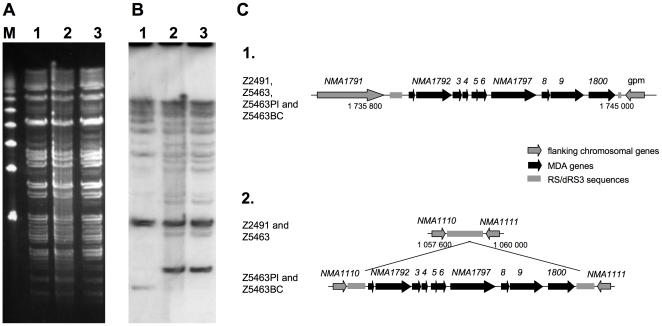
Identification of Z5463PI as the infectious strain. A- Pulsed-Field Gel Electrophoresis (PGFE) analysis using the enzyme *Bgl*II for DNA digestions, revealed by ethidium bromide. B- Southern-blot on the PFGE gel using a probe against a MDA gene (*NMA1792*). The Southern-blot is showing a second insertion of the MDA in Z5463PI and Z5463BC. M: molecular weight, 1: Z5463, 2: Z5463PI, 3: Z5463BC. C- Schematic presentation of the different insertions of the MDA in Z5463PI and Z5463BC when compared to Z2491 and Z5463. 1. Locus of the wild type insertion of the MDA (bases 1 737 566 to 1 742 107 of Z2491 genome), 2. Locus corresponding to the base 1 058 420 to 1 059 338 of Z2491, containing a second insertion of the MDA in Z5463PI and Z5463BC in a dRS3 target sequence containing a dinucleotide CT at position 1058887 of Z2491 genome.

The week before becoming ill, the patient had been working with a derivative of Z5463. Therefore, we first aimed at assessing whether the strain isolated from the blood culture was a derivative of Z5463. A PFGE analysis was performed (Fig. 1A) using material obtained directly from the frozen stocks of Z5463, Z5463PI, the isolate manipulated the week before the accident, and Z5463BC, the isolate obtained directly from the blood culture of the patient. Z5463PI had initially been selected from a colony of Z5463 for its ability to produce high amounts of the MDA phage proteins on colony immunoblots using an antibody against a peptide of NMA1796, predicted to be the main capsid protein of the MDA phage. Four passages took place between the frozen stock of Z5463 and that of Z5463PI. All three strains have an identical PFGE profile when digested by *Bgl*II (Fig. 1A), *Nhe*I or *Spe*I (data not shown). Furthermore, considering that Z5463PI had been selected on the basis of the level of production of phage proteins, a Southern-blot using an internal probe of the MDA phage was performed on the *Bgl*II digested PFGE gel (Fig. 1B). This experiment identified an additional occurence of the MDA prophage in Z5463PI and Z5463BC chromosomes. The locations of the MDA prophage in the chromosomes were mapped by LM-PCR as described in the experimental procedure section. Both strains had two copies of the MDA phage in the same places (Fig. 1C). The first copy corresponded to the initial insertion of the phage in dRS3 repeats localized between *NMA1791* and *NMA1801* (*gpm*). The second phage copy was localised in dRS3 repeats localized between *NMA1110* and *NMA1111*. The precise location of this phage was found to be exactly the same in both strains: in a dinucleotide CT at position 1058887 of the Z2491 chromosome, in the middle of the dRS3 sequence. The flanking regions of the phage occurrences sharing no homology, we concluded for an active mechanism involved in the MDA phage duplication. Altogether, these data demonstrate that the infecting strain was Z5463PI, which has two copies of the MDA prophage inserted in target dRS3 sequences.

### Z5463 has a mutator phenotype

In order to get insights into the genomic changes that may be associated with invasiveness, we undertook the sequencing of Z5463 and its two derivatives Z5463PI and Z5463BC. Whole genome sequencing was performed as described in the experimental procedure section. Some inconclusive sequences were found in the three genomes, due to frequent high homology between some neisserial genes (*opa* for instance) and duplication of some regions (mostly IS), and were further tested by PCR and sequenced.

**Figure 2:**
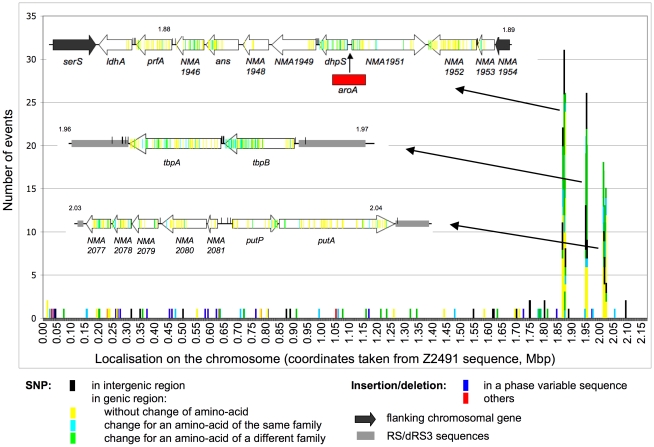
Distribution of the genetic differences (SNP and insertions/deletions) between Z2491 and Z5463. Events are classified by 1 kb sequence with Z2491 genome as reference. Each event is represented as one coloured stretch according to the nature of the difference. The three regions containing a high number of variations are highlighted: the differences are positioned on the corresponding genes represented as white arrows. Variations in the pilin locus are excluded from this figure.

As already mentioned Z2491 and Z5463 were isolated in The Gambia during the same epidemic and are therefore likely to correspond to two isolates of the same clone. We first analyzed the genomic differences between these two strains. The sequences and verifications concluded that the overall organization of the two genomes was the same. No large inversion, deletion or insertion was present in Z5463 genome when compared to that of Z2491. Differences between these two genomes are summarized in figure 2 and in Table S2. Four regions had numerous sequence differences over a range of several open reading frames. One corresponded to the pilin encoding locus with the expression site and the silent loci. The other three regions are shown in more detail in figure 2. Considering the ability of *Neisseria* to recombine with exogenous DNA, these regions of high polymorphism could correspond to recombination events that occurred following the uptake of DNA of other *Neisseria* sharing the same niche as Z5463 or Z2491. In addition to the high number of SNP detected in these regions, in one of them a gene was inserted between *NMA1950* (*dhps*) and *NMA1951*. This gene has 99% sequence identity with the phospho-2-dehydro-3-deoxyheptanoate aldolase (DAHP synthase) genes *NMW_0490* from *Nm* strain alpha275 (serogroup W135) and *NMV_0678* from *Nm* strain NMC8013 (serogroup C). All these genes contain an AroA domain; we therefore named this new gene *aroA*. This organisation, with an *aroA* homologue inserted just before *dhpS*, is observed in several *Nm* strains (serogroup C 053442 and serogroup B MC58) as well as some *Neisseria gonorrhoeae* strains (FCCP11945 and FA1090).

In addition to the above modifications, 87 localized differences were present outside these 4 regions (Fig. 2, Table 1 and Table S2). They corresponded to single nucleotide polymorphisms (SNP) or small deletions or insertions. It should be pointed out that numerous variations took place in repetitive sequences that have been described as potentially subject to phase variation. These sequences were localized either in promoter regions or in open reading frame sequences.

**Table 1:** Number and classification of the genetic events having occured between each strain.

			Z2491/Z5463	Z5463/Z5463PI	Z5463PI/Z5463BC
**deletion/insertion**	**total**		**22 (21)**	**5 (5)**	**11 (11)**
	in open reading frame	in phase variable region	13 (13)	2 (2)	9 (9)
		other	6 (5)	1 (1)	1 (1)
	in intergenic regions	phase variable region in putative promoter	3 (3)	2 (2)	1 (1)
**SNP**	**total**		**474 (66)**	**35 (18)**	**23 (16)**
	in open reading frame	without change in amino-acid sequence	276 (14)	14 (1)	8 (4)
		with change for amino-acid of the same family	65 (15)	4 (3)	4 (3)
		with change for amino-acid of a different family	86 (18)	6 (4)	10 (8)
	in intergenic regions		47 (19)	11 (10)	1 (1)
**Total**			**496 (87)**	**40 (23)**	**34 (27)**

() changes outside zones of high number of repeat.

The pilin region was excluded from analysis.

**Figure 3:**
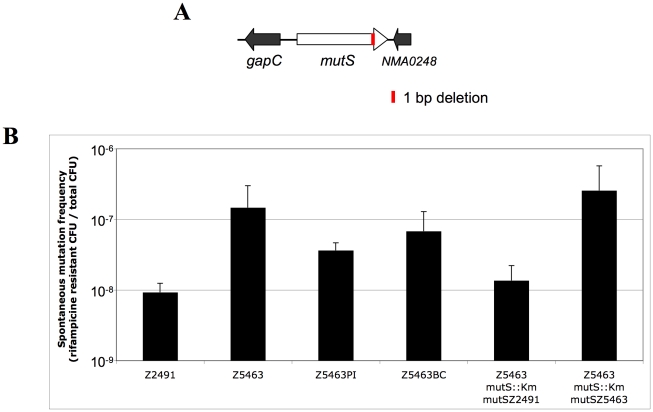
Hypermutator phenotype of Z5463, Z5463PI and Z5463BC. A- Schematic representation of the *mutS* gene of Z5463. The MutS protein of Z5463 stops at position 844 (instead of 865 aa) due to 1 bp deletion in its gene sequence leading to an early stop codon (red stretch). B- Frequency of appearance of mutants resistant to rifampicin for each of the different strains. The control strains correspond to the insertion of the *mutS* allele of Z2491 (*mutS*Z2491) or Z5463 (*mutS*Z5463) in Z5463 *mutS*::Km, selected by the spectinomycin resistance cassette.

Excluding the pilin region, the number of genes that could encode a protein with a different amino-acid sequence in Z5463 than in Z2491 is 64. In addition, 3 genes could have their expression modified by a deletion/insertion in a phase variable sequence in their promoter region. Table 2 summarizes the functional groups to which the products of these genes belong. Surprisingly, this analysis revealed the deletion of one bp at position 2478 of the *mutS* gene of Z5463, introducing a stop codon in the sequence, which would lead to the production of a truncated MutS protein (Fig. 3A). In order to address the possibility that this strain had a mutator phenotype, the ability of Z5463 to generate mutants resistant to rifampicin was assessed. Z5463 has a rate of mutations (10^−7^/generation) similar to that of a genetically engineered *mutS* mutant (data not shown). The functional *mutS* allele of Z2491 was then introduced into Z5463 as described in the experimental procedure section, which reverse this hypermutator phenotype (Fig. 3B), demonstrating that the high mutation rate observed with Z5463 was indeed due to the defective *mutS* allele.

**Table 2:** Repartition of the genes under probable phenotypical variation.

	total Z2491	comparison Z2491/Z5463	comparison Z5463/Z5463PI	comparison Z5463PI/Z5463BC
Central/intermediary/misc metabolism^a^	288^a^	7^a^	3	5
Conserved hypothetical	274	9	1	2*
Degradation of large molecules	34	2	1	0
Degradation of small molecules	22	1	0	0
Energy metabolism	96	1	0	3
Information transfer	233	5	0	0
Pathogenicity/Adaptation/Chaperonnes	96	9*	2	2
Phage/IS elements	186	8	1	0
Regulators	54	0	0	0
Surface (IM, OM, secreted…)	472	21*	5**	8
Unknown	237	4*	0	0
**total**	**1992**	**67**	**13**	**20**

a one of the gene, *aro*A, is inserted in Z5463 and its descendants but is absent from Z2491.

*one or ** two genes are submitted to phase variation due to a repeat region in their promoter region.

### Analysis of the genotypic differences between Z5463PI and Z5463BC

As for Z5463, the analysis of the sequence of Z5463PI and Z5463BC did not reveal any major genomic rearrangement when compared to that of Z2491, beside the additional insertion of the MDA phage between *NMA1110* and *NMA1111*. All the prophage sequences were identical and similar to that of the parental strain Z5463. Early hypothesis on the mechanism by which this prophage may enhance invasiveness suggested that local genomic rearrangements could occur especially around dRS3 repeats as a consequence of the expression of phage proteins. Considering that Z5463PI has been selected from Z5463 on the basis of the expression of phage proteins on colony immunoblots, we wished to test this hypothesis. Even though no genomic rearrangement had been detected by sequencing, the organisation of all the sequences surrounding dRS3 repeats, potential targets for the MDA phage, was confirmed using PCR and primers specific for each of these sequences (see the experimental procedure section for details). All sequences were amplified as expected according to the sequence of Z5463, thus excluding the possibility that insertion, deletion or inversion of sequences had occurred in Z5463PI and Z5463BC.

**Figure 4:**
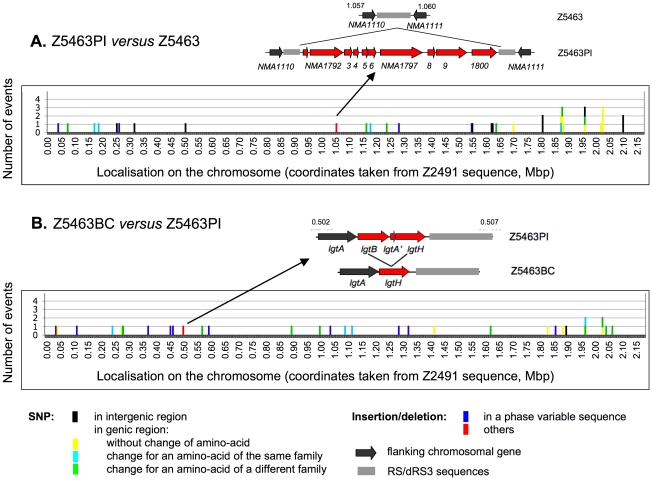
Distribution of the genetic differences between Z5463, Z5463PI and Z5463BC. Events are classified by 1 kb sequence with Z2491 genome as reference. Each event is represented as one coloured stretch according to the nature of the difference. The pilin region was excluded from this figure. A- Genetic differences between Z5463PI and Z5463. The novel insertion of the MDA is annotated as one stretched as it occurs at one specific position in the genome. B- Genetic differences between Z5463BC and Z5463PI. The region containing the deletion of *lgt*B gene is highlighted.

Unlike what has been observed between Z2491 and Z5463, comparison of Z5463, Z5463PI and Z5463BC did not reveal regions encompassing several ORFs with numerous sequence differences except for the pilin locus that is subject to antigenic variation (Fig. 4A and 4B). It should be pointed out that the *pilE* gene of Z5463BC could not be directly sequenced. Indeed various pilin variants were present in the DNA extracted directly from the blood culture (see below). In addition, one major difference between Z5463PI and Z5463BC was the deletion in Z5463BC of *lgtB* (Fig. 4B). The *lgtB* gene is adjacent to *lgtH* in Z5463 and Z5463PI and is predicted to encode a glycosyltransferase implicated in the LOS biosynthesis. Both genes have highly homologous 5′ regions. It is likely that a recombination event took place between these regions deleting the 3′ region of *lgtB*, the entire pseudogene *lgtA'* and the 5′ region of *lgtH*. This recombination led to the total deletion of *lgtB* and *lgtA'* and the restoration of a full *lgtH* gene. All other genomic differences were limited to SNP or deletions/insertion of less than 3 bp (Fig. 4, Table 1 and Table S2). 40 sequence differences were present between Z5463 and Z5463PI and 34 between Z5463PI and Z5463BC.

We next focused on those genomic modifications with possible phenotypic consequences, i.e. genes with a putative change in the encoded amino-acid sequence or genes with a phase variable promoter in which a phase variable event had occurred. Those numbers of putative phenotypic modifications are limited since respectively 11 proteins have a putative amino-acid sequence difference in Z5463PI when compared to Z5463 and 19 in Z5463BC *versus* Z5463PI. In addition two proteins have different phase variable sequences in promoter regions between Z5463 and Z5463PI and one between Z5463PI and Z5463BC. Table 2 summarizes the functional groups to which the products of these genes belong. Both isolates, like the parental strain Z5463, expressed an hypermutator phenotype due to a defective *mutS* allele (Fig. 3B). It should be pointed out that the number of changes in phase variable genes was higher between Z5463PI and Z5463BC (10 of 34 differences, 30%) than between Z5463 and Z5463PI (4 of 40 differences, 10%), thus pointing out the importance of this mechanism for *in vivo* adaptation of *Nm*. The list of the phase variable genes that have different sequences is shown Table 3. The length of the repeats responsible for phase variation of *pilC* and *opa* was variable amongst the colonies of Z5463BC, thus pointing out that a bacterial population expressing various phenotypes in these genes is probably needed to establish infection.

**Table 3:** Sequences subject to phase variation in one or more of the isolates.

	gene	Z2491	Frame	Z5463	Frame	Z5463PI	Frame	Z5463BC	Frame	References
*NMA0048*	*pglA*	(G)14	in	(G)11	in	(G)9	out	(G)10	out	[24]–[27]
*NMA0132*	hypothetical	(C)9	pro	(C)9	pro	(C)9	pro	(C)8	pro	[24], [25]
*NMA0277*	hypothetical	(A)11	pro	(A)9	pro	(A)10	pro	(A)10	pro	[24], [27]
*NMA0285/86*	hydrolase	(C)7	out	(C)8	in	(C)8	in	(C)8	in	[24]
*NMA0293*	*pilC2*	(G)11	out	(G)10	out	(G)10	out	NUS	-	[24]–[26]
*NMA0407*	acetyltransferase	(G)12	out	(G)10	in	(G)10	in	(G)9	out	[24], [25]
*NMA0475*	*hpuA*	(G)10	in	(G)9	out	(G)9	out	(G)7	in	[24], [25]
*NMA0478*	*nalP*	(C)10	in	(C)9	out	(C)9	out	(C)10	in	[24]–[27]
*NMA0609*	*pilC1*	(G)13	in	(G)10	in	(G)10	in	NUS	-	[24], [25], [27]
*NMA0619*	*wbpC*	(G)10	in	(G)11	out	(G)11	out	(G)9	out	[24], [25], [27]
*NMA0641*	conserved hypothetical	CACTCCCT(C)16	out	CACTCCCT(C)9	in	CACTCCCT(C)9	in	CACTCCCT(C)9	in	[24], [25]
*NMA0782*	phage associated	(C)7(N)10(G)7	in	(C)6(N)10(G)7	out	(C)6(N)10(G)7	out	(C)6(N)10(G)7	out	[24], [27]
*NMA0832*	glycosyltransferase	(CAAACAA)26(AT)3CTAT	in	(CAAACAA)14(AT)3CTAT	in	(CAAACAA)14(AT)3CTAT	in	(CAAACAA)14(AT)3CTAT	in	[24]
*NMA1090*	conserved hypothetical	(C)6	in	(C)6	in	(C)6	in	(C)7	out	[24]
*NMA1251*	*opcA*	(C)17	pro	(C)9	pro	(C)9	pro	(C)9	pro	[24], [25], [27]
*NMA1385*	*hsdM*	(A)8	dead	(A)8	dead	(A)9	dead	(A)8	dead	[24], [27]
*NMA1427*	glycosyltransferase	(G)9	out	(G)9	out	(G)9	out	(G)10	in	[24], [25]
*NMA1642*	*porA*	(G)12	pro	(G)12	pro	(G)11	pro	(G)11	pro	[24], [25], [27]
*NMA1792*	phage associated	(C)10(N)10(G)7	pro	(C)8(N)10(G)7	pro	(C)8(N)10(G)7	pro	(C)8(N)10(G)7	pro	[24], [25], [27]
*NMA1925*	*hmbR*	(G)10	out	(G)11	in	(G)11	in	(G)9	out	[24]–[27]
*NMA2043*	*opaA*	(CTTCT)11	out	(CTTCT)10	out	(CTTCT)10	out	NUS	-	[24], [25]

out: non functional protein;

in: functional protein;

pro: phase variation in the promoter region;

dead: stop codon in the three reading frames.

NUS: No Unique Sequence.

### Transcriptomic analysis

In order to get additional insights into modifications that could be associated with virulence, we performed a transcriptomic analysis looking for differences between Z5463 and Z5463PI on one hand, and between Z5463PI and Z5463BC on the other hand. Experiments were performed as described in the experimental procedure section and each comparison was performed 3 times. The results of each independent experiment were pooled. The only differences that were observed between Z5463 and Z5463PI were the transcription of the MDA phage genes and one gene, *kat*A, encoding the catalase.

When comparing Z5463PI and Z5463BC, several differences were observed (Table 4). Briefly, when compared to Z5463BC, Z5463PI overexpressed some of the phage proteins. This suggested that the isolate obtained from the bloodstream produced less phage than Z5463PI; this is consistent with the fact that only very small amount of circular DNA corresponding to the MDA prophage could be detected in Z5463BC (data not shown). A selection for bacterial cells not producing the MDA phage may have occurred *in vivo*. Other genes differentially regulated between the two isolates were the pseudogene *dcmH* encoding a methyltransferase, *pilT* encoding the type IV pilus retraction protein, *pdxH* encoding a pyridoxamine 5′-phosphate oxidase involved in vitamine B6 metabolic pathway and hypothetical proteins or proteins without known function. In addition, *hpuA*, encoding a haemoglobin receptor, and *nalP*, encoding an autotransporter, are overexpressed in Z5463BC when compared to Z5463PI. This is consistent with the sequencing results showing these genes to be in “ON” phase in Z5463BC and in “OFF” phase Z5463 and Z5463PI.

**Table 4:** Trancriptomic analysis of Z5463PI versus Z5463BC.

Genes	Function	PI	BC
*NMA0127*	*rplW*, 50S ribosomal protein L23		2.4
*NMA0193*	*dcmH*, cytosine-specific methyltransferase (pseudogene)	2.9	
*NMA0218*	*pilT*, type IV pilus retraction ATPase PilT	2.2	
*NMA0246*	*gapB*, glyceraldehyde-3-phosphate dehydrogenase B		2.6
*NMA0256*	putative BolA-like protein		2.8
*NMA0326*	*pyrH*, uridylate kinase		3
*NMA0328*	*rspB*, 30S ribosomal protein S2		3.36
*NMA0475*	*hpuA*, haemoglobin-haptoglobin utilization lipoprotein A		4.7
*NMA0478*	*nalP*, autotransported serine protease NalP		2.2
*NMA0737*	hypothetical membrane-associated protein	3.9	
*NMA0738*	putative transcriptional regulator	2.9	
*NMA0793*	*trmD*, tRNA (guanine-N(1)-)-methyltransferase		2.6
*NMA1060*	*dcd*, deoxycytidine triphosphate deaminase		2.8
*NMA1572*	*pdxH*, pyridoxamine 5′-phosphate oxidase	3	
*NMA1577*	conserved hypothetical protein	4.8	
*NEIMA1749*	H.8-like outer-membrane lipoprotein		2.8
*NMA1792*	putative phage replication initiation factor	7.9	
*NMA1795*	hypothetical integral membrane protein	11.8	
*NMA1837*	putative peptidase	3.2	

The numbers correspond to the fold change upregulation (average on three experiments in swap-dye) in Z5463PI when compared to Z5463BC (column PI) and in Z5463BC when compared to Z5463PI (column BC). Only the genes upregulated by more than 2-fold in at least half the experiments are mentioned here.

### Analysis of the phenotypic differences between Z5463PI and Z5463BC

Considering the above genomic differences, we analysed in more details the possible phenotypic consequences of (i) the loss of *lgtB*, (ii) the change in the phase variable sequence of the hemoglobin binding proteins and (iii) the heterogeneity in pilin sequences.

**Figure 5:**
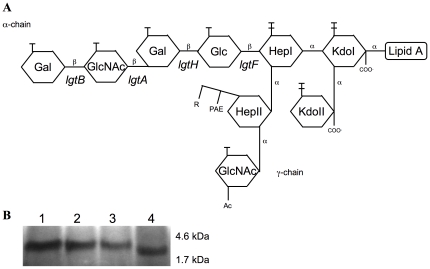
LOS variation analysis of the strain isolated from the blood culture. A- Schematic representation of the immunotype L9 LOS of serogroup A *Nm* Z2491 (adapted from Zhu and coworkers [68] and Choudhury and coworkers [52]). The deletion of the *lgtB* gene in Z5463BC is predicted to form a truncated LOS lacking the galactose on the terminal GlcNAc. B- Decreased size of the LOS from Z5463BC due to the loss of *lgtB*. Tricine SDS-PAGE gel of proteinase K LOS extracts from Z5463BC and its parental strains, revealed by silver staining. 1: Z2491 (L9 immunotype), 2: Z5463, 3: Z5463PI, 4: Z5463BC.

The L9 immunotype lipooligosaccharide (LOS) structure of Z2491 has previously been described [52] and is schematically presented in figure 5A. According to the sequences and the apparent size of their LOS (Fig. 5B), Z5463 and Z5463PI express the same LOS as Z2491. On the other hand, Z5463BC expresses a truncated LOS as seen in figure 5B. This result is consistent with the absence of *lgtB* that adds a galactose on the terminal GlcNAc of the LOS. LOS structure has been described to be important for human serum resistance. We further tested the capacity of serum resistance of Z5463BC by incubating the strain with 25% of human serum. No differences were seen between Z5463BC and Z5463PI or Z5463 using commercial serum or serum isolated from the patient (data not shown). The three strains grew normally in these sera in contrast to a mutant lacking capsule which was killed by complemented serum.

**Figure 6:**
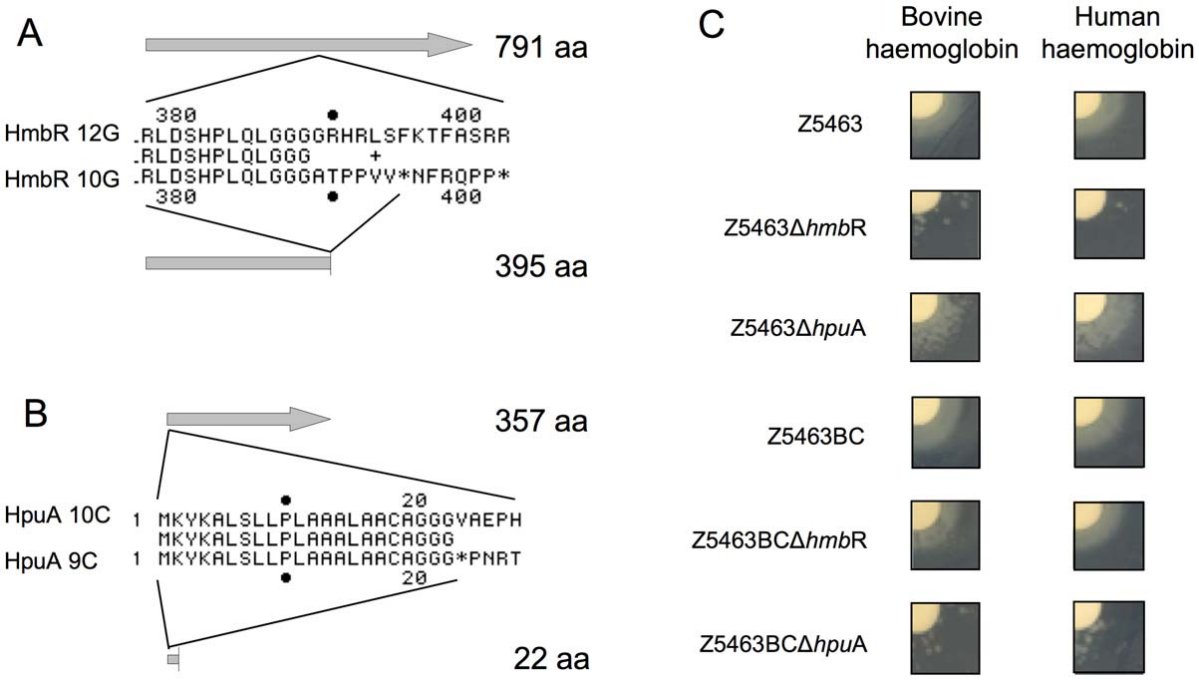
Ability of the blood culture strain to use human and bovine haemoglobin. A- Sequences and alignment of the HmbR protein. 12G in Z5463BC lead to a full-length protein; 10G in Z5463 and Z5463PI is responsible for a stop codon at aa 396. B- Sequences and alignment of the HpuA protein. 10C in Z2491, Z5463 and Z5463PI lead to a full-length protein; 9C in Z5463BC is responsible for a stop codon at aa 23). C- Growth in iron depleted medium and supplemented with disks containing human or bovine haemoglobin.

Among the differences in phase variable genes, we concentrated on those important for iron acquisition *in vivo*. *Nm* can acquire iron *in vivo* either *via* transferrin binding proteins TbpA and TbpB [53], or *via* receptors specific for haemoglobin-haptoglobin [15], [16], [54]. Two sets of genes are required for the latter, *hmbR* on one hand and *hpuA*/*hpuB* on the other hand. Surprisingly, *hmbR* is turned OFF in Z5463BC (Fig. 6A), whereas the *hpuA* gene is ON (Fig. 6B) which is the exact opposite of Z5463 and Z5463PI. To confirm that these genotypic modifications were consistent with the phenotype expressed by these variants, insertion mutants were engineered by introducing the *cat* gene, conferring resistance to chloramphenicol, in *hpuA* and the spectinomycin resistance cassette in *hmbR*. The strains were then tested for their ability to use human or bovine haemoglobin. As shown in figure 6C, Z5463Δ*hmbR* cannot grow correctly on haemoglobin whereas Z5463Δ*hpuA* has no growth defect. Z5463PI mutants had the same phenotype (data not shown). In contrast, Z5463BCΔ*hpuA* was unable to grow properly on haemoglobin unlike Z5463BCΔ*hmbR*. Altogether these observations clearly confirm that chelating iron from the haemoglobin/haptoglobin is important for *in vivo* growth of *Nm*, and that during the *in vivo* passage the HpuAB pathway has been selected in preference to the HmbR pathway.

**Figure 7:**
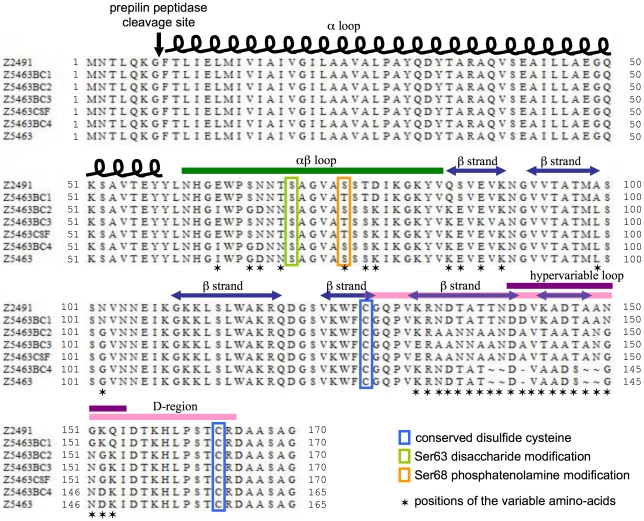
Sequences of the various pilin variants isolated *in vivo*. Several sequences were isolated from the blood culture in contrast to the only variant isolated from its parental strains passed *in vitro*. The alignment of the different *pilE* sequences shows 4 different variants of the PilE protein found in the blood culture and differing from the one found in the CSF of the patient. Sequence alignments of full-length prepilin are shown with highlights on structural features (inspired by Craig and corworkers [69]).

Considering that the pilin locus could not be sequenced due to heterogeneity of the pilin variants expressed in the bacterial population isolated from the bloodstream, eight colonies were isolated from Z5463BC and the pilin locus of each of these isolated colonies sequenced, thus identifying four different pilin variants. Furthermore a PCR amplifying the pilin locus was performed on Z5463BC without starting from an isolated colony and cloned in *E. coli*, eight of these clones were sequenced, they identified an additional two pilin variants in addition to the above four (Fig. 7). In addition, a PCR was performed directly on a sample of the CSF because no bacteria grew out of the CSF sample due to early antibiotic administration. This PCR was cloned in *E. coli* and the *pilE* gene sequenced from ten of these clones. Interestingly a single pilin variant was present in the CSF, and this variant was different from those isolated in the bloodstream.

**Figure 8:**
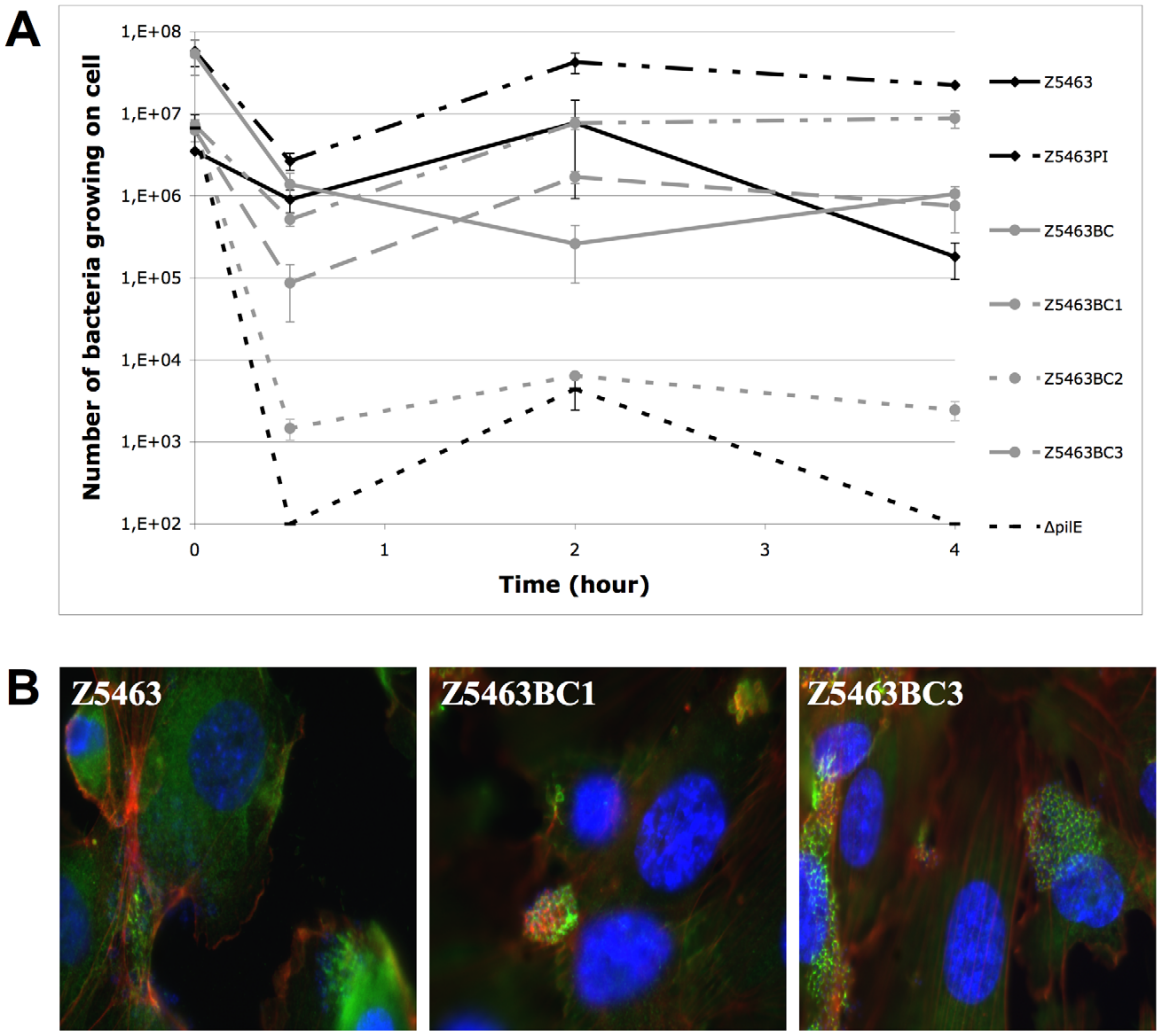
Phenotype on cells of the different pilin variants of the blood culture strain. A. Kinetic of adhesion of the different pilin variants of Z5463BC and Z5463 on hCMEC/D3 cells. An isogenic mutant of Z5463 lacking PilE was used as a negative control (Δ*pilE*). B. Signalling properties of the different pilin variants of Z5463BC, capable of adhesion on hCMEC/D3 cells, were assessed by immunofluorescence: in blue DAPI stain, in red actin, in green phalloïdin. All the variants recruit cell actin and phalloïdin under over 95% of the bacterial colonies forming a typical cortical plaque.

Type IV pili is an essential bacterial attribute allowing interaction of capsulated meningococci with endothelial cells. Different pilin variants allow the bacteria to express different phenotypes of bacterial-cell interaction. Among the four pilin variants directly isolated from colonies of Z5463BC, one has a non-piliated phenotype (Z5463BC2) and among the three other sequences, two were identical at the protein level. The three different derivatives were assessed for their ability to interact with cells (Fig. 8A) and to signal to cells (Fig. 8B). Both piliated isolates adhered to human brain microvessel endothelial cells (hCMEC/D3) and induced the recruitment of focal adhesion plaques with an index of recruitment superior to 95% (Fig. 8 B; data not shown). No major difference was observed between these variants except that they had varying abilities to form microcolonies onto the apical surface of the cells.

## Discussion

This work describes the characterization of a laboratory strain causing meningitis in a previously healthy research worker. The knowledge of events leading to the disease allowed us to assess the number of bacterial division that occurred *in vivo*. We hypothezised that the number of SNP between Z5463 and Z5463PI per generation that has occurred between Z5463 and Z5463PI was identical to the number of SNP between Z5463PI and Z5463BC per generation between Z5463PI and Z5463BC. The number of generations between Z5463PI and Z5463BC corresponds to the number of generation *in vivo* and in the blood culture flask. We could approximate the latter by knowing the time it took for the flask to be detected as positive in the clinical microbiology laboratory and determining the doubling time of *Nm* in these flasks. The number of generation between Z5463 and Z5463PI was approximated knowing the number of *in vitro* passages that had been performed. According to the above, the number of bacterial generations *in vivo* is roughly 25. This number is only an approximation as it implies that the frequency of SNP between strains passed on plate or in human remain the same and is directly dependent on the number of bacterial generations. Surprisingly, the number of *in vivo* generations remains limited.

The high number of SNP and phase variation detected in the descendants of Z5463 (calculated mutation rate of about 10^−7^ per generation, data not shown) even with few generations is in accordance with its hypermutator phenotype due to a non-functional MutS protein [55]. Several serogroup A strains isolated from epidemics have already been shown to display a hypermutator phenotype [31], [55], a functional MutS or MutL can complement some of these mutations. The three strains, Z5463, Z5463PI and Z5463BC, contain a mutated allele conferring a non-functionnal MutS. Nevertheless we detected more differences in phase variable genes during the *in vivo* passage than *in vitro* (respectively 30% and 10% of the total differences). Interestingly, most of the proteins subject to phase variation between the three strains are related to virulence. For instance, the NalP protein [56] is expressed in Z5463BC but not in Z5463 and Z5463PI. This autotransporteur modulates the secretion of itself, App and IgA protease [57] as well as LbpB [58] and is therefore believed to be important for virulence. Furthermore, different antibodies against these proteins are found in patient sera [59], their release could help to escape the host immune system. Other surface components are indirectly affected, the *pgl*A gene is OFF in Z5463BC preventing the glycosylation of pilin on serine 63, which is not the case in the parental strains; this change would not affect piliation or adhesion to human cell [60] but can confer an advantage to the bacteria to escape the immune system. Furthermore, *hmbR* is turned OFF in Z5463BC, whereas *hpuA* is ON which is the opposite of Z5463 and Z5463PI. We investigated the haemoglobin utilisation systems in Z5463BC and showed that both HmbR and HpuA were functional when in the “ON” phase. This switch between the haemoglobin receptors may be a means to escape the host defences because of the presence in the patient blood of antibodies directed against HmbR. Unfortunately, we were unable to test the presence of specific antibodies in the patient blood directly after the infection.

It appears that the hypermutator phenotype due to the MutS protein confers sequence variations highly impacted *in vivo* by the selective pressure imposed by the host immune system. This was expected as the infecting strain is a derivative of an epidemic clone and therefore can be considered as having all the necessary attributes to be potentially fully adapted and virulent to its host. For instance, Z5463 and Z5463PI both possess a functional haemoglobin utilisation system, but each human individual has a different and evolving immune system repertoire, which imposes a high selective pressure on the bacteria.

For some phase (*opa*, *pilC*) and antigenic (pilin) variable genes, we obtained various sequences in the blood isolate. This apparent polymorphism of bloodstream strains has previously been documented in patients for *opa* and pilin expression [61]. The *pilE* gene from Z5463PI was identical to that of Z5463 showing no selection *in vitro*. One of the six different *pilE* variants of Z5463BC shared the same sequence as Z5463. The other variants were certainly due to recombination with different *pilS* sequences of Z5463. We were unable to find the CSF pilin variant among the different variants we isolated from the blood culture. Similar results on pilin variation during *in vivo* studies have already been observed for *N. gonorrhoeae*. Gonococcal pilin antigenic variation has been shown *in vitro* [62] and *in vivo* by studying the piliation state of bacteria infecting the same family or by following infection in volunteers [63]. These studies showed the higher rate of switch *in vivo* when compared to *in vitro* [63]. The presence of a pool of bacteria in the patient blood could increase the ability of the bacteria to invade and survive in its host.

Surprisingly, no recombination events could be detected in Z5463PI or Z5463BC despite the presence of a second MDA prophage in their genome. A recent epidemiological study had demonstrated that the presence of the prophage was associated with increased invasiveness in adolescents [21]. The phage does not encode any known virulence factors and deletion of the entire phage was without effect in several laboratory models of meningococcal pathogenesis [20]. This phage is always inserted into small repeats of the meningococcal genome, designated dRS3. This suggests that some phage proteins have the ability to recognize these sequences. In addition, one of its proteins (the product of *NMA1800*) has homologies with the IS110/492 family of transposases. One hypothesis regarding the role of this phage is that, when expressed, some of its proteins could promote genomic rearrangements with possible phenotypic consequences, especially on invasiveness [20], [51], [64]. The fact that a duplication of the prophage had occurred while selecting for isolates producing high amount of phage proteins, reinforced this hypothesis. This duplication is restricted to the phage, the sequences on each side of the two insertions do not have any homology, thus demonstrating that the element is active and capable of reintegrating itself in *Nm* genome. This duplication of the MDA was stable upon passages (the MDA being exactly in same location between Z5463PI and Z5463BC) but was not associated with genome rearrangement.

The differences observed in the bloodstream strain (SNP, phase variation and localised recombination of single genes) have been selected over a very short period of time. A larger number of generation elapsed between the clones (isolated in different patients) lead to a predominance of recombination events as between Z2491 and Z5463 that were isolated from the same epidemics at the same time.

A surprising result was the deletion of *lgtB*, which is involved in lipooligosaccharide synthesis [65]. Z2491 expresses a well-described L9 immunotype LOS [52], as do Z5463 and Z5463PI. Z5463BC expresses a truncated LOS and we can assume that this changes its immunoreactivity. All isolates were resistant to complemented commercial sera and to the patient sera (sampled 2 years post infection). A truncated L3-immunotype LOS lacking the terminal galactose, due to the deletion of *lgtB*, still produces a bactericidal antibody response in mice similarly to the wild type non-truncated LOS [66]. Furthermore, these antibodies react with LOS but not with lacto-N-neotetraose as do antibodies elicited by the wild-type strain. It is possible that the recombination between *lgtB* and *lgtH* has been selected due to the presence in the patient blood of antibodies specific for the terminal structure of the LOS.

To conclude, this study shows that the sequence variations *in vivo*, occurring during a limited number of bacterial generations, give rise to important phenotypic changes in the bacteria and have a direct impact on the adaptation of the bacteria to its host. This illustrates perfectly the potential of *Nm* to adapt its repertoire of genes therefore becoming more fit to overcome the host immune system and increasing its virulent in a new host [67].

## Supporting Information

Table S1 **List of the oligonucleotides used in this study.**
(XLS)

Table S2 **List of the genes containing a genetic event leading to an amino-acid change in the protein, potentially affecting the phenotype of one of the strain.**
(XLS)
